# IL-17A Neutralization Improves the Neurological Outcome of Mice With Ischemic Stroke and Inhibits Caspase-12-Dependent Apoptosis

**DOI:** 10.3389/fnagi.2020.00274

**Published:** 2020-09-04

**Authors:** Qingqing Dai, Song Han, Ting Liu, Jiayin Zheng, Cui Liu, Junfa Li, Shujuan Li

**Affiliations:** ^1^Department of Neurology, Beijing Chao-Yang Hospital, Capital Medical University, Beijing, China; ^2^Department of Neurobiology and Center of Stroke, Beijing Institute for Brain Disorders, School of Basic Medical Science, Capital Medical University, Beijing, China; ^3^Department of Neurology, Beijing Luhe Hospital, Capital Medical University, Beijing, China

**Keywords:** interleukin-17A neutralization, neuronal apoptosis, oxygen–glucose deprivation (OGD), middle cerebral artery occlusion (MCAO), casepase-12

## Abstract

We previously reported that the levels of astrocyte-derived interleukin-17A (IL-17A) increased both in the peri-infarct region and cerebrospinal fluid (CSF) of mice with 1-h middle cerebral artery (MCA) occlusion/12-h reperfusion (1-h MCAO/R 12 h)-induced ischemic stroke. However, the effects of IL-17A neutralization on the neurological outcome of mice with ischemic stroke and its underlying molecular mechanism are unclear. In this study, we found that the intracerebroventricular injection of IL-17A-neutralizing monoclonal antibody (mAb; 2.0 μg) could reduce the infarct volume, alleviate neuron loss, and improve the neurological outcomes of mice with 1-h MCAO/R 24-h- or 3-day-induced ischemic-stroke mice. The IL-17A neutralization could also significantly inhibit the increase of pro-caspase-3 cleavage through caspase-12-dependent cell apoptosis, as well as preventing the decrease of antiapoptotic factor B-cell lymphoma 2 (Bcl-2) and the increase of proapoptotic Bcl-2-associated X protein (Bax) in the peri-infarct region of mice following ischemic stroke. In addition, we confirmed that the recombinant mouse (rm) IL-17A could significantly aggravate 1-h oxygen–glucose deprivation/24-h reoxygenation (1-h OGD/R 24 h)-induced ischemic injuries in cortical neurons in a dose-dependent manner, and the rmIL-17A could also exacerbate neuronal apoptosis through caspase-12 (not caspase-8 or caspase-9)-dependent pathway. These results suggest that IL-17A neutralization could improve the neurological outcome of mice with ischemic stroke through inhibiting caspase-12-dependent neuronal apoptosis.

## Introduction

Stroke is the second most common cause of death worldwide and a major global cause of disability (Feigin et al., 2014). In China, stroke has already been the major leading cause of death and disability (Johnston et al., 2009; Liu et al., 2011). With the update guideline for ischemic stroke, more patients could benefit from the intravenous alteplase and mechanical thrombectomy within the window time (Powers et al., 2019). However, there were still many limitations that lead to some patients losing the chance for treatment. There were more than 250 clinical trials, including more than 1,000 brain-protective molecules researches that have failed. Thus, it is crucial to look for new therapeutic targets for acute ischemic stroke (O’Collins et al., 2006).

As we know, immune response plays a pivotal role after ischemic stroke, and immune interventions were highlighted in recent years. Inflammatory factors (Fu et al., 2015), including interleukin-1β (IL-1β), interferon-γ, IL-17, matrix metallopeptidase-9, and C-C motif chemokine 2, could up-regulate cell adhesion molecule expression and promote accumulation of the leukocyte to the surface of cerebral blood vessels, which led further neural structure damage directly or indirectly (Seifert et al., 2014; Fu et al., 2015). Among the inflammatory cytokines, IL-17A was reported to aggravate secondary tissue damage after ischemic stroke (Wang et al., 2009; Zhang et al., 2014; Li S. et al., 2017). After onset of ischemic stroke, together with tumor necrosis factor-α, IL-17 exacerbated neutrophil infiltration. It was also found that interruption of the IL-23/IL-17 cascade could decrease infarct size and improve neurological outcome after ischemic stroke (Arunachalam et al., 2017; Gelderblom et al., 2018). The effects of IL-17A and anti-IL-17A treatment have also been proved in asthma, rheumatoid arthritis, multiple sclerosis, transplant rejection, and inflammatory bowel disease (Chen and Kolls, 2017).

Our previous study demonstrated that IL-17A remarkably increased both in the peri-infarct region and cerebrospinal fluid (CSF) of mice after 1-h middle cerebral artery occlusion/12-h reperfusion (1 h MCAO/R 12 h)-induced ischemic stroke, and the immunofluorescent staining also indicated that astrocytes were the main source of IL-17A in the brain of mice with ischemic stroke (Li S. et al., 2017). Furthermore, we identified that IL-17A could alleviate astrocyte apoptosis through inhibiting the downregulation of IL-17RA and -17RC membrane translocations under ischemic condition (Dai et al., 2019). As reported, there are three general pathways of apoptosis. The extrinsic pathway was associated with the cleavage of pro-caspase-8, whereas the cleaved caspase-9 mediated the mitochondrial pathway and cleaved caspase-12 triggered the endoplasmic reticulum (ER) pathways (Broughton et al., 2009). However, which cell apoptosis pathways that triggered by IL-17A is unclear during the development of ischemic stroke. In this study, we further explored the potential therapeutic effect of IL-17A-neutralizing monoclonal antibody (mAb) on the neurological outcome of mice with ischemic stroke and its underlying molecular mechanism of neuronal apoptosis.

## Materials and Methods

### MCAO/R-Induced Ischemic Stroke Mouse Model

Adult male C57BL/6J mice at age of 6–8 weeks (weighing 18–23 g) were purchased from the Jackson Laboratory (Bar Harbor, ME, USA) and maintained in the Experimental Animal Center of Capital Medical University, People’s Republic of China. The mice were housed in the temperature-controlled (23°C ± 2°C), humidity-controlled (40–70%), and 12-h light–dark cycle room with access to food and water *ad libitum*. All procedures were performed in accordance with the recommendations in the Guide for the Care and Use of Laboratory Animals of the National Institutes of Health and approved by the experimental animal ethics committee of Capital Medical University (SCXK2016–0006).

In total, 70 of C57BL/6J mice were randomly divided into three groups, and detailed information is listed in Table 1. The data collectors/investigators were blinded to the grouping during evaluating the neurological deficits and behavioral tests/or analyzing the data. The MCAO/R-induced ischemic stroke model was prepared and performed as previous research (Wei et al., 2016; Li X. et al., 2017). First, the mice were anesthetized with 1% pentobarbital sodium [70 mg/kg, intraperitoneally (i.p.)]. Second, the common carotid artery (CCA) and the external carotid artery (ECA) were exposed with a ventral midline incision. A permanent ligation was made at the distal portion of the ECA, and a temporary ligation was made at the CCA. A vascular clip was used in the internal carotid artery (ICA) to restrict the blood flow; and then a 4–0 surgical nylon monofilament with its tip (0.23 mm in diameter) was inserted to the ICA from the small incision of ECA. When the monofilament reached the middle cerebral artery (MCA), the mild resistance was touched, and the length of monofilament was inserted in it about 11.0–12.0 mm. After occluding for 1 h, after removing the monofilament, the incision of ECA was permanently ligated and recovered the blood flow from the CCA. For monitoring cerebral blood flow (CBF) during MCAO surgery, laser Doppler flowmetry was conducted with the PeriFlux 5000 system (Perimed, Stockholm, Sweden). Regional CBF decreased by 80% in mice after surgery and recovered completely 1 h later after removing monofilament. Postoperative mice were fed in a warm cage for a minimum of 2 h. The IL-17A-neutralizing mAb or immunoglobulin G (IgG) isotype was injected from the contralateral ventricle within 3 h.

**Table 1:** Animal distribution and usage.

Groups	TTC	WB	BT	IF	Death	Mortality (%)
**Sham**	6	6	0	0	0	0 (0/12)
1 h MCAO/R 24 h + IgG isotype	6	6	0	0	0	0 (0/12)
1 h MCAO/R 24 h + IL-17A-mAb	6	6	0	0	1	7.7 (1/13)
**Sham**	0	0	10	6	0	0 (0/10)
1 h MCAO/R 3 d + IgG isotype	0	0	10	6	1	9.1 (1/11)
1 h MCAO/R 3 d + IL-17A-mAb	0	0	10	6	2	16.7 (2/12)
**Total** (70)	18	18	30		4	5.7 (4/70)

*MCAO, middle cerebral artery occlusion; R, reperfusion; TTC, triphenyl tetrazolium chloride; WB, Western blot; IF, immunofluorescent staining; BT, behavior test. The numbers of IF were included in the BT*.

### Intracerebroventricular Injections of IL-17A-Neutralizing mAb

Mice were randomly divided into three groups: sham, MCAO + IgG isotype, and MCAO + IL-17A mAb groups. The IL-17A-neutralizing mAb (2.0 μg, #560268, Becton, Dickinson and Company, NJ, USA) was injected within 3 h after MCAO, with the mouse IgG isotype injection as control (#MAB002, R&D System, MN, USA). The intracerebroventricular (i.c.v) injection procedure was performed according to the previous research (Bu et al., 2011). Mice were anesthetized with sodium pentobarbital (70 mg/kg, i.p.) and placed in a stereotaxic frame. The point of injection was in the right cerebral ventricle using the following coordinates: 0.5 mm posterior to Bregma, 1.0 mm lateral to bregma, and 3.2 mm below the skull surface. After injection, the mice were fed in warm cages with food and water to recover for 24 h or 3 days.

### Evaluation of Neurological Deficits and Behavioral Tests

The MCAO-treated mice were tested at 24 h and 3 days after reperfusion. Neurological deficits were measured using the simplified procedure created by Rodriguez et al. (2005), with the detailed criteria listed as follows: 0, no neurological dysfunction; 2, slight dysfunction in mobility and presence of passivity; 4, moderate neurological dysfunction; 6, more handicapped animals with more marked hypomobility, circling, tremor, jerks and/or convulsions, forelimb flexion and moderate motor incoordination; 8, respiratory distress and total incapacity to move/coordinate; and 10 refers to death due to 1-h MCAO/1- to 3-day reperfusion. If the level of deficits did not meet the precise criteria, the nearest appropriate number 1, 3, 5, 7, or 9 was recorded.

At the same time, neurological deficits were also evaluated using the modification of Longa scoring system as follows: 0, no neurological deficit; 1, failure to extend left forepaw fully; 2, circling to the left; 3, falling to the left; and 4, did not walk spontaneously and had a depressed level of consciousness (Roof et al., 2001).

Corner test was tested as described before (Li et al., 2004). The mouse was put in a corner at an angle of 30 degrees in front of the nose. Contact with the vibrissae led to a rear and the direction in which the mouse turned was recorded. There is no turning preference in the normal mice. But the ischemia mice prefer to turn to the nonimpaired side. The index of right turns was calculated for 20 times in each test.

The wire hanging was used to test the motor function of mice with a wire, which was hanged on 25-cm height above soft bedding ground. The mouse was placed on the wire and only let the forepaws grasp the wire. The time that the mouse spent hanging was recorded. A score of 0 was given to mice that fell immediately; 300 s was the time-out period. Each mouse was trained 3 days before surgery and tested three times every testing day. The average time was calculated.

### Measurements of Infarct Volume and Edema

The 1-h MCAO mice were sacrificed at 24 h of reperfusion, and the brain was isolated on the ice and cut into 1.0-mm-thick coronal sections. Brain sections were incubated for 20 min in a solution of 2% triphenyl tetrazolium chloride (TTC; Sigma–Aldrich, St. Louis, MO, USA) at 37°C. As reported by Wexler et al. (2002), the images of stained slices were scanned, and the infarct volume was calculated by Image Pro Plus 6.0 (Media Cybernetics, Rockville, MD, USA). Edema ratio (E) was calculated using the equation: *E* = (ΣVL − ΣVR)/(ΣVL + ΣVR) × 100%; ΣVR and ΣVL are the volume of right (nonischemic) and left (ischemic) hemisphere, respectively. Background was calculated using the following equation: *B* = ΣVS/ΣVT × 100%, where ΣVS is the volume of the unstained white matter in the sham group and ΣVT is the total brain volume. Considering the effect of edema and background, infarct volume was determined indirectly and represented as a percentage of the entire brain using the equation: I = [ΣVI × (1 − E)/ΣVT × (1 − B)] × 100%, where ΣVI is the volume of tissue that is not stained with TTC in MCAO mice.

### Primarily Cultured Cortical Neurons

Neurons were obtained from cortexes of postnatal C57BL/6J mice as the following process. After removing the meninges and blood vessels, the cortex was cut into pieces with 1.0 mm^3^, digested with trypsin–EDTA (0.25%) for 10 min, and beat to single-cell suspension fluid. And then the cells were cultured onto plates in Dulbecco modified eagle medium (DMEM, G11995500BT, Gibco, Beijing, China), which contained 10% horse serum (16050-122, Gibco, Grand Island, NY, USA), 10% fetal bovine serum (10099-141, Gibco), 1% penicillin–streptomycin solution (15070063, Life Technologies, Carlsbad, ON, Canada), and 0.25% L-glutamine (25030-081, Gibco) for 4 h. After culturing for 4–6 h, the medium was changed with the neurobasal medium (21103-040, Gibco), which contained 2% B27 supplement (17504-044, Gibco) that was changed completely every 72 h. At the seventh day, the neurons on the six-well (1 × 10^6^ cells) and 96-well (1 × 10^4^ cells) plates (5% CO_2_ + 95% O_2_) were prepared for Western blot analysis and 3-(4, 5-dimethylthiazol-2-yl)-5(3-carboxymethonyphenol)-2-(4-sulfophenyl)-2H-tetrazolium (MTS) assay, respectively. The experiment was repeated six times under the same culture conditions.

### Oxygen-Glucose Deprivation/Reoxygenation-Induced Cell Ischemia *in vitro*

According to our previous protocol (Li S. et al., 2017), the primary cortical neurons were cultured to mature (7 days). For the condition of oxygen–glucose deprivation (OGD), the medium was changed with the glucose-free DMEM medium and cultured in a hypoxic chamber with the mixture gas of 5% CO_2_ + 2% O_2_ + 93% N_2_; After 1-h OGD, the reoxygenation condition (R, 5% CO_2_ + 95% O_2_) was recovered with neurobasal medium containing 2% B27 for 24 h. The incubator was kept at 37°C with saturated humidity.

### Recombinant Mouse IL-17A Treatments

Recombinant mouse IL-17A (rmIL-17A, 421-ML-025/CF, R&D System) was respectively dissolved in the medium at final concentrations of 0.5, 1.0, 10,100, 250, and 500 ng/ml. When the neurons were treated with 1-h OGD/R 24 h, they were cultured in glucose-free DMEM with different concentrations of rmIL-17A and hypoxic condition (5% CO_2_ + 2% O_2_ + 93% N_2_) for 1 h, and then the neurobasal medium containing 2% B27 with different dose of rmIL-17A was recovered to supply with glucose and oxygen (5% CO_2_ + 95% O_2_) for 24 h. Finally, the neurons were collected and used for the next experiments.

### MTS Assay

The matured neurons were seeded on the 96-well plates and treated with rmIL-17A in a dose-dependent manner for 24 h in a humidity-saturated incubator at 37°C at normoxic (5% CO_2_ + 95% O_2_) or 1-h OGD/R 24 h conditions. Then each plate was added 20 μl reagent of the Cell Titer 96^®^ Aqueous kit (G3580, Promega, WI, USA) and cultured for another 1 h at normoxic condition. After that, microplate reader was used to measure the absorbance at 490 nm.

### Western Blotting Analysis

For detection of the cleavage levels of caspase-3, caspase-8, caspase-9, and caspase-12 and the relative expression levels of Bax and Bcl-2 in the peri-infarct region, the brains of mice were removed at 24 h of reperfusion after 1-h MCAO, and the cerebral cortex of peri-infarct region was separated. The corresponding regions of the sham group and the IgG isotype groups were also collected. Similarly, the cleavage levels of caspase-3, caspase-8, caspase-9, and caspase-12 were also analyzed in primarily cultured cortical neurons. The neurons and the tissues were thawed and homogenized with buffer C [50 mM Tris-Cl, pH 7.5, containing 2 mM EDTA; 1 mM EGTA; 100 mM iodoacetamide (SH-group blocker); 5 μg/ml each of leupeptin, aprotinin, pepstatin A, and chymostatin; 50 mM potassium fluoride; 50 nM okadaic acid; 5 mM sodium pyrophosphate + 2% sodium dodecyl sulfate (SDS)]. Protein concentration was determined by using BCA kit (23225 Pierce Company, Rockford, IL, USA) with albumin in Buffer C as standards.

Protein samples (30 μg per lane) were separated on 12% SDS–polyacrylamide gel electrophoresis and then transferred onto polyvinylidene difluoride (PVDF) membranes (GE Healthcare, Little Chalfont, UK). The transferred PVDF membrane was blocked with 10% skimmed milk in Tween-20/Tris-buffered salt solution for 1 h at room temperature. The membranes were then incubated overnight at 4°C with the primary antibodies as follows: rabbit monoclonal anti-caspase-3 (1:1,000; #9665s, CST, Danvers, MA, USA), rabbit monoclonal anti-caspase-8 (1:1,000; #C4106, Sigma–Aldrich, Merck, Germany), mouse monoclonal anti-caspase-9 (1:1,000; #9508S, CST), rat monoclonal anti-caspase-12 (1:1,000; #C7611–2ML, Sigma–Aldrich), rabbit monoclonal anti-Bcl-2 (1:1,000; CST), rabbit monoclonal anti-Bax (1:1,000; #5023, CST), and mouse mAb against β-actin (1:10,000, 60008-1-Ig, Proteintech Corp., IL, USA). The secondary antibodies include the horseradish peroxidase (HRP)-conjugated goat anti-rabbit IgG (1:4,000; Stressgen Biotechnologies Corporation, Victoria, BC, Canada), rabbit anti-mouse IgG (1:4,000; The Jackson Laboratories), and HRP-goat anti-rat IgG (1:2,000, AS028; Abclonal, Wuhan, China). The Enhanced Chemiluminescence kit (GE Healthcare) and Fusion FX (Vilber, French) were used to detect the signals.

The ImageJ (Java, National Institutes of Health, Baltimore, MD, USA) was used for Western blot quantitative analysis. The band density was calculated as follows: the band density of cleaved caspase/(procaspase + cleaved caspase)/corresponding β-actin, as well as the band density of Bcl-2 or Bax/their corresponding β-actin, and then were normalized by using their respective sham groups.

### Immunofluorescent Staining

At the third day of reperfusion after 1-h MCAO, mice were anesthetized with 1% pentobarbital sodium (70 mg/kg, i.p.) and perfused transcardially with 0.9% NaCl for 3 min followed by 4% paraformaldehyde in 0.1 M phosphate-buffered saline (PBS), pH 7.4, for 30 min. The brains were separated quickly and fixed with 4% paraformaldehyde at 4°C for 24 h. And then the brains were dehydrated in 20% and 30% sucrose solution for 24 h, respectively, at 4°C. The brains were mounted in optimal cutting temperature compound and frozen in liquid nitrogen for 5 min and frozen in the −80°C for 24 h. The cryoprotected brains were sectioned into 20-μm-thick slices in the coronal plane through the entire rostrocaudal regions using a microtome (CM1950 Clinical Cryostat; Leica Biosystems, Germany).

For immunofluorescent staining, brain slices were first incubated in the 8% goat serum with PBST buffer (0.5% Triton X-100 in 0.1 M PBS) for 2 h at room temperature. The primary mouse mAbs against NeuN (1:300, ab104224; Abcam, Boston, MA, USA) were applied at 4°C overnight. After washing three times (10 min each time) with PBST buffer, the slices were incubated in the Alexa Fluor 488 goat anti–mouse IgG (1:250, A11029; Molecular Probes, Thermo Fisher Scientific, Rockford, IL, USA) for 2 h at room temperature. Finally, the slices were mounted with 2-(4-amidinophenyl)-6-indolecarbamidine dihydrochloride-containing mounting media. The microphotographs were taken bilaterally with Leica microscope. There were three views chosen randomly from each image. The number of NeuN staining positive cells of every view was counted by ImageJ software. And the average number of per image was used for statistical analysis.

### Statistical Analysis

All measuring data were represented as mean ± SEM. As the attributes data, including neurological score, Longa score, laterality index, and latency to fall were expressed as quartile. Statistical analysis was performed as follows: two-way analysis of variance (ANOVA) for MTS results; one-way ANOVA for the results of the Western blot, the immunofluorescent staining with NeuN, and the behavior tests. The followed multiple comparisons were conducted by using Bonferroni with the software GraphPad Prism version 7.2 for Mac (GraphPad Software, La Jolla, CA, USA). *p* < 0.05 was considered as statistically significant.

## Results

### Effect of IL-17A Neutralization on Neurological Outcome of Mice With Ischemic Stroke

**Figure 1:**
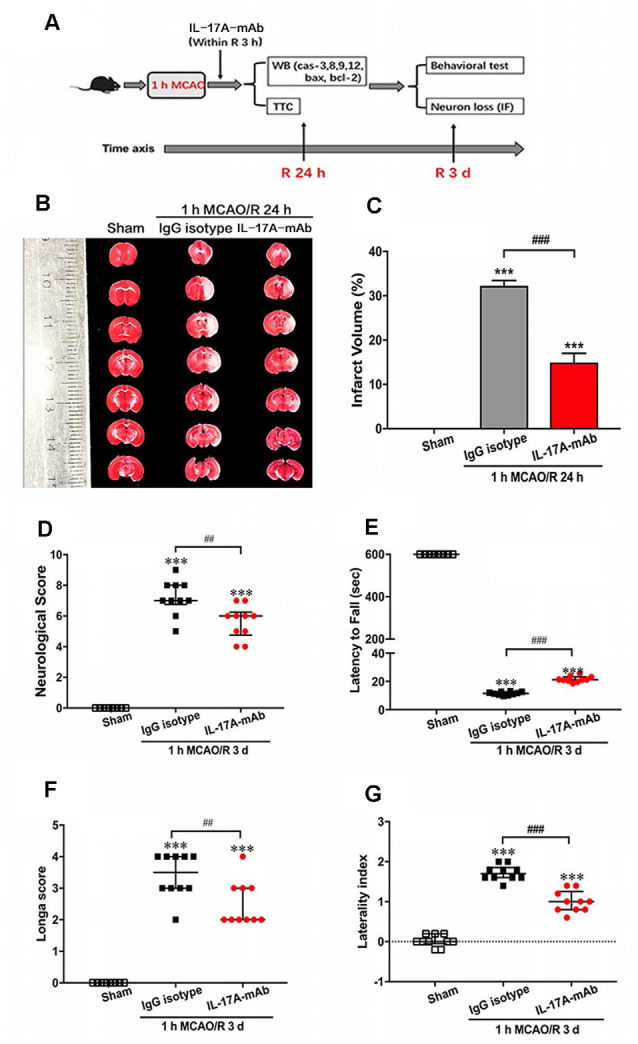
Interleukin-17A (IL-17A)–neutralizing monoclonal antibody (mAb) improved the neurological outcome of mice with ischemic stroke. **(A)** The diagram of experimental design showed that the intracerebroventricular injection of IL-17A-neutralizing mAb (2 μg, i.c.v) or IgG isotype within 3-h reperfusion after 1-h MCAO treatment. At 24 h of reperfusion, the mice were sacrificed for Western blot and triphenyl tetrazolium chloride (TTC) staining. At 3 days of reperfusion, the mice were used to evaluate the neurological function and then sacrificed for immunofluorescent staining. **(B)** At 24 h of reperfusion after 1-h MCAO, the infarct size of IL-17A-neutralizing mAb group was significantly smaller than the IgG isotype group. **(C)** Bar graph showed the statistical analysis results of infarct size among three groups (*n* = 6 per group). Data are presented as mean ± SEM; ***p < 0.001 vs. sham group; ^###^*p* < 0.001 vs. 1-h MCAO/R 24 h + IgG isotype group. In addition, the IL-17A-neutralizing mAb could significantly improve the neurological function that included neurological score **(D)**, the latency to fall of wire hanging **(E)**, the Longa score **(F)**, and the laterality index of corner test **(G)** of mice following 1 MCAO/R 3 days (*n* = 10 per group). Data are presented as quartile; ***p < 0.001 vs. *s*ham group; ^##^*p* < 0.01, ^###^*p* < 0.001 vs. 1-h MCAO/R 24 h + IgG isotype group.

Our previous research indicated that IL-17A production increased both in the CSF and serum of ischemic-stroke mice that contributed to ischemic injury (Zhang et al., 2014; Li S. et al., 2017). In addition, IL-17A level increased earlier in the CSF than the serum. In this study, the potential therapeutic effects of IL-17A-neutralizing mAb (i.c.v) were evaluated in 1-h MCAO/R 24 h or 3 days *in vivo*. The time axial and experimental design is shown in Figure 1A. At 24 h of reperfusion after MCAO, the TTC staining results showed that the infarct volume of IL-17A-neutralizing mAb-treated mice was obviously smaller than that of IgG isotype-treated mice (Figures 1B,C; *p* < 0.001, *n* = 6 per group). Moreover, at the third day of reperfusion after 1-h MCAO, the neurological score (Figure 1D, *p =* 0.0015) and Longa score (Figure 1F, *p* = 0.0048) after IL-17A-neutralizing mAb treatment were significantly lower than those of the IgG isotype group. The corner and wire hanging test results indicated that IL-17A-neutralizing mAb could improve the motor function compared with that of IgG isotype-treated mice with ischemic stroke (Figures 1E,G; *p* < 0.001, *n* = 6 per group). These results suggest that IL-17A neutralization could improve the neurological outcomes of mice with ischemic stroke.

### Effect of IL-17A Neutralization on Neuron Loss in Peri-infarct Region of Mice With Ischemic Stroke

**Figure 2:**
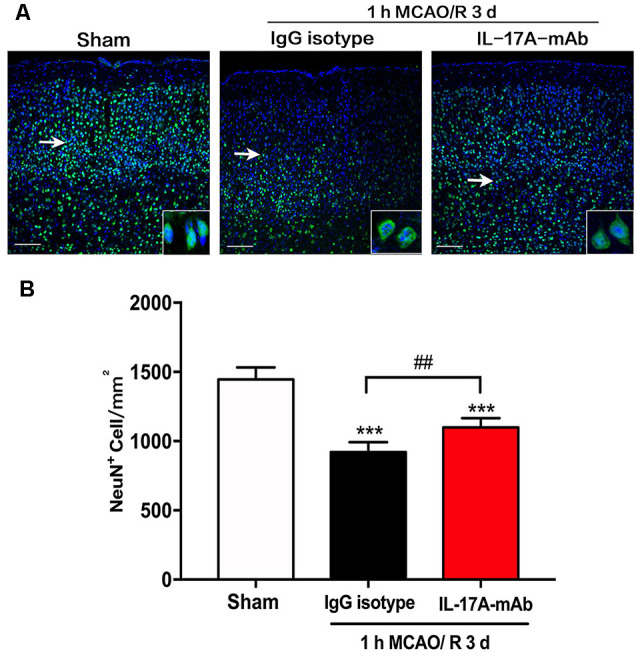
Effect of IL-17A-neutralizing mAb on neuron loss in peri-infarct region of mice with ischemic stroke. **(A)** The representative immunofluorescent staining results showed the effect of IL-17A-neutralizing mAb on NeuN positive numbers in peri-infarct region of mice following 1-h MCAO/R 3 days’ treatment (scale bar = 100 μm). **(B)** Statistical analysis results demonstrate that the i.c.v. injection of IL-17A-neutralizing mAb (2.0 μg) could significantly inhibit the NeuN positive cell loss when compared with that of 1-h MCAO/R 3 days + IgG isotype group. Data are presented as mean ± SEM; ***p < 0.001 vs. sham group; ^##^*p* < 0.01 vs. 1-h MCAO/R 3 days + IgG isotype group (*n* = 6 per group).

The immunofluorescent staining with neuron-specific marker NeuN was used in the sham, IgG isotype, and IL-17A mAb groups at the third day of reperfusion after 1-h MCAO. The results showed that 1-h MCAO/R 3-day-induced ischemic stroke led to a significant neuron loss (Figures 2A,B; *p* < 0.001, *n* = 6 per group) in the peri-infarct region of IgG isotype and IL-17A mAb-treated mice. However, IL-17A-neutralizing mAb could obviously alleviate the neuron loss in the peri-infarct region (Figures 2A,B; *p* = 0.0028, *n* = 6 per group) when compared with that of IgG isotype group. These results demonstrate that early application of IL-17A-neutralizing mAb could effectively reduce the neuron loss in the peri-infarct region of mice with ischemic stroke.

### IL-17A-Neutralizing mAb Alleviated Caspase-12-Dependent Apoptosis in Peri-infarct Region of Mice With Ischemic Stroke

**Figure 3:**
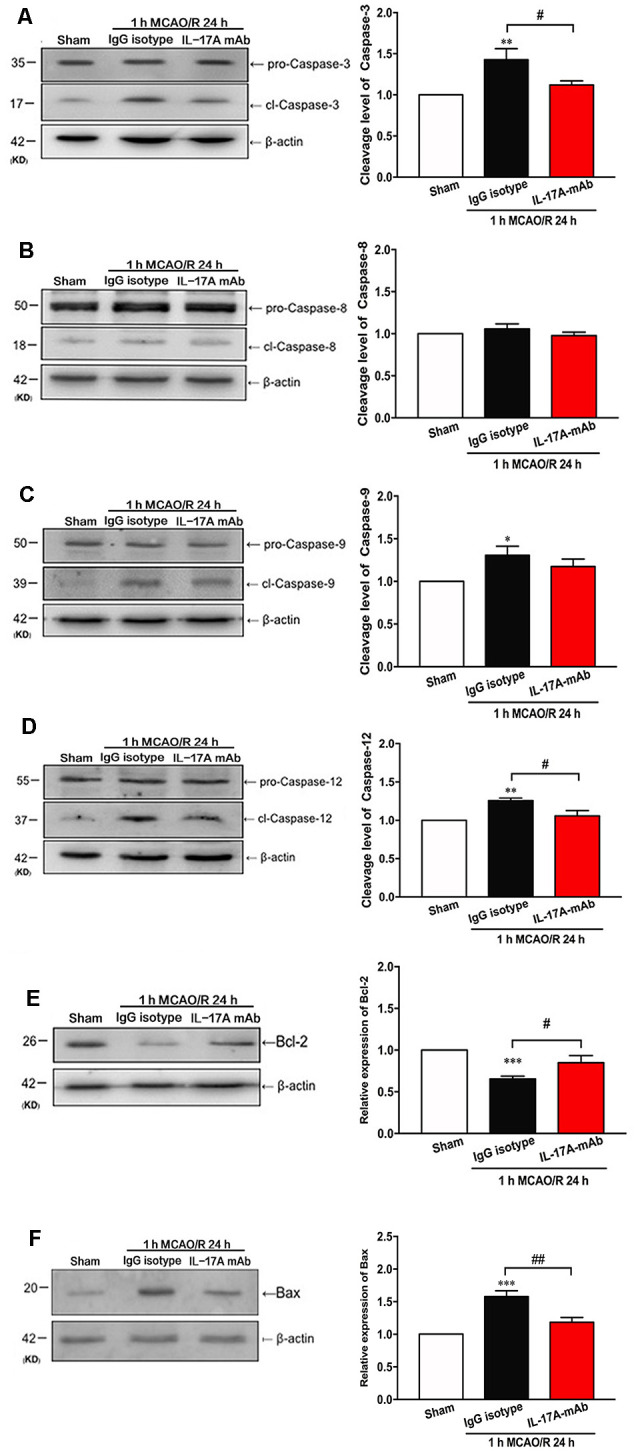
IL-17A-neutralizing mAb alleviated the neural cell apoptosis in the peri-infarct region of mice with ischemic stroke through caspase-12-dependent pathway. The representative and quantitative analysis results of Western blot showed that the i.c.v. injection of IL-17A-neutralizing mAb (2.0 μg) could significantly inhibit the cleavage levels of caspase-3 **(A)** and caspase-12 **(D)** in the peri-infarct region of mice following 1-h MCAO/R 24 h. In addition, the injection of IL-17A-neutralizing mAb could significantly increase Bcl-2 expression levels **(E)**, but decrease the expression levels of Bax **(F)** in the peri-infarct region of mice with ischemic stroke. However, the injection of IL-17A-neutralizing mAb did not affect the cleavage levels of caspase-8 **(B)** and caspase-9 **(C)** in the peri-infarct region of mice after 1-h MCAO/R 24-h treatment. Data are presented as mean ± SEM; *p < 0.05, **p < 0.01, ***p < 0.001 vs. sham group; ^#^*p* < 0.05, ^##^*p* < 0.01 vs. 1-h MCAO/R 24 h + IgG isotype group (*n* = 6 per group).

Over the last decade, the role of cell apoptosis in ischemic stroke has been proven (Miles and Knuckey, 1998; Broughton et al., 2009). To explore the detailed pathways of IL-17A-triggered cell apoptosis, the cleavage levels of caspase-3, caspase-8, caspase-9, and caspase-12 and the expression levels of B-cell 2 (Bcl-2) and Bcl-2 associated X protein (Bax) were determined in the peri-infarct region of mice with ischemic stroke *in vivo*. The cleavage levels of caspase-3 (Figure 3A; *p* = 0.0402, *n* = 6 per group) and caspase-12 (Figure 3D; *p* = 0.0124, *n* = 6 per group) decreased obviously in the 1-h MCAO/R 24 h + IL-17A mAb group compared with that of the 1-h MCAO/R 24 h + IgG isotype group. The similar tendency was observed in the expression levels of Bax (Figure 3F, *p* = 0.017, *n* = 6 per group). The cleavage level of caspase-9 increased in the 1-h MCAO/R 24 h + IgG isotype group (Figure 3C, *p* = 0.0299, *n* = 6 per group), but no significant changes of caspase-9 cleavage level were observed between the IgG isotype and IL-17A mAb groups. However, there were no significant changes of caspase-8 cleavage among the three groups (Figure 3B). In addition, the injection of IL-17A-neutralizing mAb could significantly increase Bcl-2 expression levels (Figure 3E, *p* = 0.0414, *n* = 6 per group), but decrease the expression levels of Bax (Figure 3F, *p* < 0.01, *n* = 6 per group) in the peri-infarct region of mice with ischemic stroke. These results indicated that IL-17A-neutralizing mAb could alleviate caspase-12-dependent neural cell apoptosis in the peri-infarct region of mice with ischemic stroke.

### rmIL-17A Aggravated Caspase-12-Dependent Neuronal Apoptosis After 1-h OGD/R 24 h Treatment

**Figure 4:**
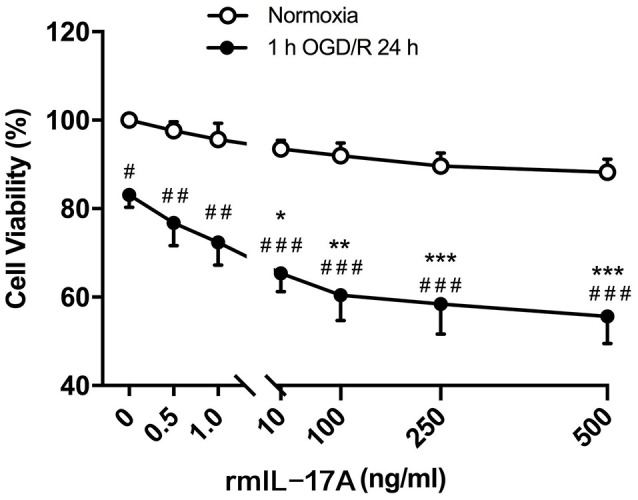
Effect of rmIL-17A on the cell viabilities in primarily cultured cortical neurons following 1-h oxygen-glucose deprivation/reoxygenation (OGD/R) 24-h treatment. The results of 3-(4, 5-dimethylthiazol-2-yl)-5(3-carboxymethonyphenol)-2-(4-sulfophenyl)-2H-tetrazolium (MTS) assay showed that rmIL-17A could aggravate the cell viabilities of the primarily cultured cortical neurons with dose-dependent manner after 1-h OGD/R 24-h treatments (*n* = 6 per group). Data are presented as mean ± SEM; *p < 0.05, **p < 0.01, ***p < 0.001 vs. 1-h OGD/R 24 h and 0.0 ng/ml rmIL-17 treatments; ^#^*p* < 0.05, ^##^*p* < 0.01, ^###^*p* < 0.001 vs. corresponding normoxic group (*n* = 6 per group).

To confirm the effect of IL-17A on ischemic neurons, the cell viability was determined in primarily cultured cortical neurons under the conditions of 1-h OGD/R 24 h and rmIL-17A treatment. The results in Figure 4 showed that the cell viabilities significantly decreased in 1-h OGD/R 24 h-treated cortical neurons when compared with that of their corresponding normoxic group (*p* < 0.05, 0.01, or 0.001; *n* = 6 per group). The exogenous addition of rmIL-17A could dose-dependently aggravate the ischemic injuries of cortical neurons compared with their corresponding 0.0 ng/ml rmIL-17A treatment (*p* < 0.05, 0.01, or 0.001; *n* = 6 per group). However, the rmIL-17A treatments (0.5–500 ng/ml) did not affect the cell viabilities of cortical neurons under normoxic condition (*n* = 6 per group).

**Figure 5:**
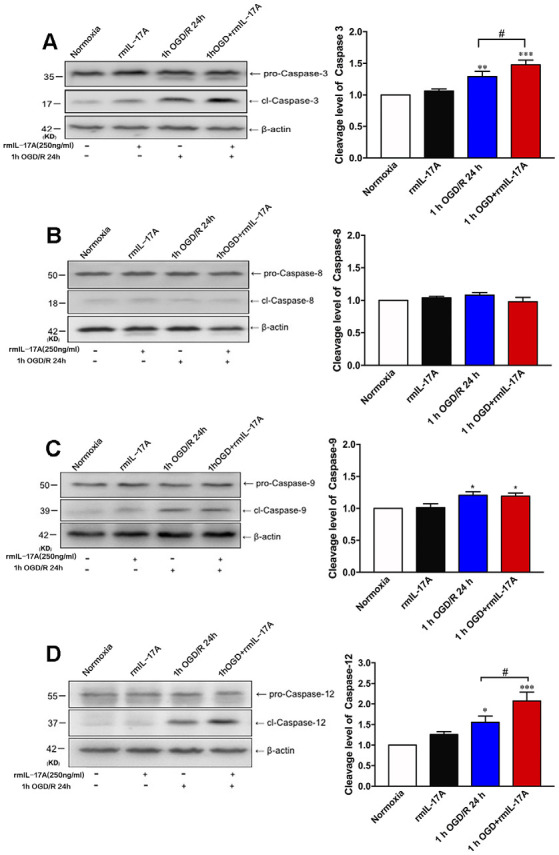
Effect of rmIL-17A on cell apoptosis in 1-h OGD/R 24-h-treated primary cortical neurons. The representative and quantitative analysis results of Western blot demonstrated that rmIL-17A (250 ng/ml) could remarkably exacerbate the cleavage levels of caspase-3 **(A)** and caspase-12 **(D)** in 1-h OGD/R 24*-*h-treated cortical neurons. However, the rmIL-17A treatment did not affect the cleavage levels of caspase-8 **(B)** and caspase-9 **(C)** in primary cortical neurons under the condition of 1-h OGD/R 24 h. Data are presented as mean ± SEM; *p < 0.05, **p < 0.01, ***p < 0.001 vs. normoxic group; ^#^*p* < 0.05 vs. 1-h OGD/R 24-h group (*n* = 6 per group).

To further determine which pathway of cell apoptosis was affected by rmIL-17A, the cleavage levels of caspase-3, caspase-8, caspase-9, and caspase-12 were detected in primary cortical neurons after 1-h OGD/R 24 h and rmIL-17A (250 ng/ml) treatments. In Figure 5, the typical Western blot and quantitative analysis results showed that the cleavage levels of caspase-3 (Figure 5A, *p* = 0.078), caspase-9 (Figure 5C, *p* = 0.0215), and caspase-12 (Figure 5D, *p* = 0.0266) significantly increased in cortical neurons following 1-h OGD/R 24 h treatment (*n* = 6 per group). Furthermore, the addition of rmIL-17A (250 ng/ml) could exacerbate 1-h OGD/R 24 h-induced cleavages of caspase-3 (Figure 5A, *p* = 0.0329) and caspase-12 (Figure 2D, *p* = 0.0386), but not caspase-9 (Figure 5C) in cortical neurons (*n* = 6 per group). In addition, the caspase-8 cleavage was not observed in cortical neurons after 1-h OGD/R 24 h with or without rmIL-17A treatment (Figure 5B, *n* = 6 per group). These results suggest that rmIL-17A could aggravate the ischemic injuries of cortical neurons through caspase-12-dependent cell apoptosis.

## Discussion

Inflammatory response, excitotoxicity, apoptosis, and a series of complex cascades were involved in the process of pathophysiology after ischemic stroke (Li Y. et al., 2017). As the energy-dependent cell death program, studies indicated that there were many apoptotic neurons in the peri-infarct region after several hours or days of stroke onset (Culmsee et al., 2005; Taylor et al., 2008). Evidences also indicated that the expression of IL-17A increased both in the brains of mice and humans after ischemic stroke and was one of the most important inflammatory cytokines (Li et al., 2005; Wang et al., 2009). The research suggested that IL-17A aggravated ischemia/reperfusion injury *via* increase in calpain-mediated TRPC6 proteolysis (Zhang et al., 2014). The IL-17A deficiency mice showed smaller infarct size than those of wild type after MCAO (Shichita et al., 2009). In this study, we found that IL-17A could aggravate neuronal apoptosis through caspase-12-dependent apoptosis pathway *in vivo* and *vitro*. Meanwhile, IL-17A-neutralizing mAb could effectively rescue the neuronal apoptosis of peri-infarct region in the ischemic-stroke mice. These findings are coincident with the result that IL-17A-neutralizing mAb after stroke could reduce the neutrophil infiltration, which led to the recovery of neurological function in MCAO mice (Gelderblom et al., 2012).

In this study, we found that in the peri-infarct region of MCAO mice, the cleavage levels of caspase-3, caspase-9, and caspase-12 increased remarkably. However, IL-17A neutralization only inhibited the cleavage levels of caspase-3 and caspase-12 (not caspase-9). It suggested that IL-17A-neutralizing mAb would reduce apoptosis through caspase-12-mediated ER-stress pathway. Studies have identified that ER-stress-induced cell apoptosis was involved in central nervous system disease, cancer, cardiovascular diseases, and metabolic diseases (Groenendyk et al., 2013; Roussel et al., 2013; Rieusset, 2017). Caspase-12 mainly located on the ER, which accumulated the unfolded protein and led to the disequilibrium of calcium homeostasis (Wang and Kaufman, 2016). In this study, we also found that rmIL-17A aggravated the cell death of 1-h OGD/R 24 h-treated cortical neurons through the pathway of caspase-12-dependent cell apoptosis.

As an inflammatory cytokine, IL-17A was expressed by various cell types including T_H_17 cells, γδT cells, some nonlymphocytic cells, and even astrocytes in the ischemic brain to cause the inflammatory responses (Dessalle et al., 2016; Ksiazek-Winiarek et al., 2017; Li S. et al., 2017). Recently, Qiu et al. (2017) found that low dose of apelin-36 in MCAO rats could inhibit ischemia/reperfusion-induced expression of CCAAT/enhancer binding protein homologous protein (CHOP) and 78-kDa glucose-regulated protein (GRP78), improving the neurological outcomes by alleviating the apoptosis (Wang and Kaufman, 2016). In addition, Bcl-2 family was involved in both mitochondria and the ER by translocation of Ca^2+^ to the mitochondria (Hadley et al., 2018). It was summarized in the review that CHOP could increase the expression level of Bax and prevent the expression level of Bcl-2 (Ksiazek-Winiarek et al., 2017). Su et al. (2011) suggested, GRP78-mediated ER-stress could down-regulate the expression level of Bcl-2, which mediates mitochondria-dependent cell death (Hadley et al., 2018). In agreement with these studies, in the *in vivo* experiments, we found that IL-17A-neutralizing mAb treatment could effectively improve the expression of antiapoptotic factor Bcl-2 and inhibit proapoptotic factor Bax expression. Therefore, we suggested that CHOP might be the effective target of IL-17A-neutralizing mAb in prevention of ischemic/reperfusion injury. The detailed mechanism should be further identified in the next research project.

In conclusion, we found that IL-17A could aggravate the cell death of cortical neurons under ischemic condition through the pathway of caspase-12-dependent cell apoptosis *in vitro*. With application of the IL-17A-neutralizing mAb, it also identified that IL-17A exacerbates cell apoptosis in peri-infarct region and through affecting the caspase-12-dependent pathway. IL-17A-neutralizing mAb could effectively reduce the infarct volume and neuron loss in the peri-infarct region and then improve the neurological outcomes of mice with ischemic stroke *in vivo*. These findings may provide the proof that inhibiting the level of IL-17A could be the therapeutic target for alleviating the caspase-12-dependent neuronal apoptosis in ischemic stroke.

## Data Availability Statement

The raw data supporting the conclusions of this article will be made available by the authors, without undue reservation.

## Ethics Statement

The animal study was reviewed and approved by experimental animal ethics committee of the Capital Medical University (SCXK2016-0006).

## Author Contributions

QD performed the research. SL designed the research study. SH and JL contributed essential reagents and tools. TL, JZ and CL analyzed the data. QD wrote the manuscript. SL and JL reviewed and edited the manuscript.

## Conflict of Interest

The authors declare that the research was conducted in the absence of any commercial or financial relationships that could be construed as a potential conflict of interest.
